# SPORTS INJURIES AMONG AMATEUR ATHLETES AT A BRAZILIAN UNIVERSITY

**DOI:** 10.1590/1413-785220172502165651

**Published:** 2017

**Authors:** André Marangoni Asperti, Tiago Lazzaretti Fernandes, André Pedrinelli, Arnaldo José Hernandez

**Affiliations:** 1 Universidade de São Paulo, Faculdade de Medicina, Hospital das Clínicas, Instituto de Ortopedia e Traumatologia, São Paulo, SP, Brasil.

**Keywords:** Athletic injuries, Epidemiology, Ankle, Anterior cruciate ligament

## Abstract

**Objective:**

To obtain information on the incidence and nature of sports injuries at a Brazilian university.

**Method:**

Data from 396 student amateur athletes (61% male) playing 15 different sports during the 2013 season were retrospectively evaluated. Subjects completed the National Collegiate Athletic Association Injury Surveillance System questionnaire at the conclusion of the 2013 sports season. Injuries that resulted in at least one day of time lost were included. Exposure was defined as one student amateur athlete participating in one practice or game and is expressed as an athlete-exposure (A-E).

**Results:**

Injury rates were significantly greater in games (13.13 injuries per 1000 A-Es, 95% CI = 10.3-15) than in practices (4.47 injuries per 1000 A-Es, 95% CI = 3.9-5.1). The mechanisms that accounted for the most injuries in games and practices were player contact (52.9%) and non-contact (54.5%), respectively. Ankle ligament sprains were the most common injury (18.2% of all reported injuries). A relatively high incidence of anterior cruciate ligament injury was also observed (0.16 injuries per 1000 A-Es).

**Conclusion:**

Brazilian student amateur athletes are at great risk of sustaining non-contact injuries such as ankle sprains and anterior cruciate ligament injuries. ***Level III of Evidence, Study of non consecutive patients; without consistently applied reference ''gold'' standard.***

## RESUMO

**Objetivo:**

Obter a incidência e as características das lesões esportivas em atletas de uma universidade do Brasil.

**Métodos:**

Os dados de 396 atletas amadores universitários (61% homens) de 15 modalidades, referentes ao ano de 2013, foram avaliados retrospectivamente. Os atletas responderam o questionário ISS (Injury Surveillance System) adotado pela NCAA (National Collegiate Athletic Association) no final da temporada esportiva de 2013. Foram incluídas as lesões que resultaram em pelo menos um dia de afastamento. A exposição foi definida como um atleta amador universitário participando de um treino ou jogo e foi expressa como uma exposição-atleta (E-A).

**Resultados:**

As taxas de lesões em jogos (13,13 lesões por 1000 E-A, 95% IC = 10,3 - 15) foram significantemente maiores do que em treinos (4,47 lesões por 1000 E-A, 95% IC = 3,9 - 5,1). Os mecanismos envolvidos na maioria das lesões em jogos e em treinos foram contato com outro jogador (52,9%) e sem contato (54,5%), respectivamente. A torção de tornozelo foi a lesão mais comum (18,2% entre todas as lesões). Observou-se alta incidência de lesões do ligamento cruzado anterior do joelho (0,16 lesões por 1000 E-A).

**Conclusão:**

Os atletas amadores universitários brasileiros têm maior risco de sofrer lesões sem contato, como torção de tornozelo e lesão do ligamento cruzado anterior. ***Nível de Evidência III, Estudo de pacientes não consecutivos; sem padrão de referência "ouro" aplicado uniformemente.***

## INTRODUCTION

Since 1982, The American National Collegiate Athletic Association (NCAA) has supported an Injury Surveillance System (ISS), which collects injury and exposure data from 16 sports.^1^ Over time, the data collected from ISS turned to be one of the most important source of knowledge in the sports medicine field.

One of the most serious sports injuries, anterior cruciate ligament (ACL) tear, had its mechanism and gender distribution elucidated by ISS by a 5 year study with basketball and soccer players from NCAA as subjects.^2^ In addition to orthopedics and sports medical areas, other ones were supported by data from ISS. The prevalence of sudden cardiac death^3^ and the effects of sports related concussions in collegiate athletes,^4^ both topics of increasing interest in the literature, were addressed by ISS and published in journals of great impact. 

Data regarding sports injuries have resulted in numerous successful injury prevention initiatives, including new models of football helmets to protect players from concussions^5^ and equilibrium exercises to prevent ankle sprains in volleyball and basketball players.^6^ This is in agreement with the 4 step injury prevention model proposed by van Mechelen et al.,^7^ in which we: (1) identify the problem, (2) establish etiology and mechanisms, (3) develop, evaluate, and implement interventions, and (4) reevaluate the effect via continued surveillance. Sports injuries preventive measures have improved across the years. The "American Academy of Orthopaedic Surgeons" (AAOS) and the "American College of Sports Medicine" (ACSM) currently support neuromuscular training in girls who play soccer to help reduce the rate of ACL injury.^8^ Besides, recently, evidence of the efficacy of such programs in male soccer players have also been found.^9^ The purpose of this study is to get information on the incidence and on the nature of injuries student athletes get in sports practicing at a Brazilian University. In the future, this study may allow adoption of injury prevention strategies similar to those implemented by the NCAA's ISS.^1^

## METHODS

The study participants were 427 student amateur athletes who were official graduating students from either the Medical School or from The Physical Education School of the same Brazilian university who were practicing at least one of the 15 sport modalities offered by these schools in 2013. Athletes who were not official graduating students were excluded (e.g., athletes who had already graduated). An appropriate institutional review board approved the project (CAPPesq 513.548 - 22/01/2014) and each participant provided written informed consent prior to participation. The study is in accordance with the Helsinki Declaration of 1975, which was revised in 1983. Data composed of exposures and injuries regarding the 2013 season were collected retrospectively by the adoption of NCAA's ISS questionnaire.^1^ The athletes answered the questionnaire after the last practice or game of the 2013 season. (Appendix 1) 

A reportable injury had to meet the following criteria: (1) injury occurred as a result of participation in a university practice or game in 2013 and (2) injury resulted in restriction of the student-athlete´s participation or performance for one or more days beyond the day of injury.^1^ An exposure was defined as one athlete participating in one practice or one game (athlete-exposure, A-E).^1^

Quantitative data concerning exposure in games was obtained by summing up the number of athletes who took part in each game in 2013. The quantitative data concerning exposure in practicing was obtained by multiplying the total number of student athletes by the number of practicing sessions in 2013, and afterwards, subtracting the number of absences from the result of the multiplication. Both game and practicing exposure data were calculated, separately, for each type of sport. The calculations were based on the ISS exposure report.^1^ All the information necessary to obtain the exposure data was provided by the athletes by answering a questionnaire.

A retrospective analysis was carried out after injury and exposure data compilation. Outcomes included game and practice injury rates (both overall and by sport), injury mechanism (non-contact, other contact, player contact and unknown), the distribution of injuries by body part (head and neck, upper extremity, trunk and back, lower extremity and other system), and the rates of select injuries (ankle ligament sprains and anterior cruciate ligament) by sport. Injury rates were expressed as the number of injuries per 1000 A-Es,^10^ with a confidence interval of 95%. Data regarding injury mechanism and the distribution of injuries by body part were determined by percentages.

## RESULTS

### Sample characteristics

Among the 427 student athletes included in the study, 396 (92.8%) answer the questionnaire and so participated as subjects. Among those subjects, 241 (60.9%) were men and 155 (39.1%) were women, with an overall mean age of 24.15 (±5.63) years old. Table 1 shows the distribution of athletes across 15 sports modalities. Most sports, including indoor soccer, handball, volleyball, basketball, athletics, swimming, table tennis, karate and tennis have both men´s and women´s teams. Rugby, judo and water polo include only men´s teams, while softball is composed of only a women´s team.

**Table 1:** Number and percentage of athletes by sport.

Sports	Number and percentage of athletes
Indoor Soccer	73 (18.4%)
Handball	69 (17.4%)
Volleyball	59 (14.8%)
Basketball	56 (14.1%)
Soccer	45 (11.3%)
Rugby	31 (7.8%)
Athletics	30 (7.5%)
Softball	23 (5.8%)
Water Polo	21 (5.3%)
Swimming	20 (5%)
Baseball	16 (4%)
Judo	13 (3.2%)
Table Tennis	10 (2.5%)
Karate	10 (2.5%)
Tennis	6 (1.5%)

Note: The sum of percentages is more than 100% due to the fact that 22.7% of student-athletes played two modalities.

### Injuries Rates

Among the 396 subjects who answered the questionnaire, 228 (57.6%) suffered at least one injury. Among those who suffered at least one injury, 68% (156) suffered just one injury, 23% (52) suffered two injuries, 8% (17) suffered three injuries and 1% (3) suffered four injuries. Altogether, in 2013, 59,491 exposures and 323 injuries were totaled.

Across all sports, the game injury rate (13.13 per 1000 A-Es, 95% CI = 10.3 - 15) was 2.93 times higher than the practice injury rate (4.47 per 1000 A-Es, 95% CI = 3.9 - 5). These rates equate to one injury every four games and one injury every 10 practices for a team of 20 participants. 

**Figure 1:**
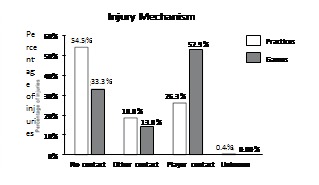
Distribution (percentages) of injuries by injury mechanism for practice and games for 15 sports in 2013. Player contact = contact with another competitor; Other contact = contact with playing surface, apparatus, ball or with other in environment (e. g., wall, fence, spectators); No contact = no apparent contact (rotation about planted foot) or no apparent contact (other).

Overall practice and game injury mechanisms are shown in Figure 1. The mechanism that accounted for the majority of injuries in games was player contact (52.9%) and in practice was non-contact (54.5%).

**Figure 2:**
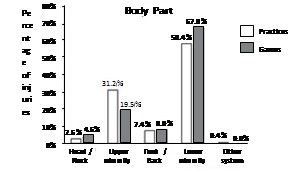
Distribution (percentages) of injuries by body part for games and practices for 15 sports in 2013.

The overall distribution of injuries by body part is shown in Figure 2. In both practices and games, more than 50% of all reported injuries were in the lower extremity. The ankle (18.2%) and knee (11.2%) accounted for the most injuries. 

**Figure 3:**
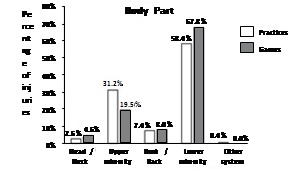
Overall (A) game and (B) practice injury rates by sport in 2013.

Game and practice injury rates by sport are shown in Figure 3A-B. For games, rugby had the highest rate of injury (42.42 per 1000 A-Es) and athletics had the lowest (3.97 per 1000 A-Es). For practice, judo had the highest rate of injury (13.47 per 1000 A-Es) and swimming had the lowest (0.81 per 1000 A-Es). Swimming, tennis and karate presented injuries only in practice, while table tennis did not present any practice or game injuries.

### Rates of Select Injuries (Ankle Ligament Sprains and Anterior Cruciate Ligament Injuries) by Sport

Table 2 and 3 show the frequency, distribution and rates of select injuries (ankle ligament sprains and ACL injuries, respectively). Ankle ligament sprains were reported 59 times. These injuries accounted for approximately one quarter of all injuries in soccer, volleyball and indoor soccer. Soccer (2.33 per 1000 A-Es) and volleyball (2.11 per 1000 A-Es) had the highest rates of ankle ligament sprains. Regarding ACL injuries, 10 injuries were reported. Basketball (0.45 per 1000 A-Es) and handball (0.38 per 1000 A-Es) had the highest rates. (Table 3) 

**Table 2:** Frequency, distribution, and rates of ankle sprains in games and practice combined in 2013.

Ankle ligament sprains	Frequency	Percentage of all injuries	Injury rate per 1000 athleteexposures	95% Confidence interval
Soccer	11	26.8	2.33	0.9 - 3.7
Volleyball	9	25.0	2.11	0.7 - 3.5
Rugby	6	19.3	1.98	0.4 - 3.6
Judo	2	14.2	1.86	-0.8 - 4.5
Indoor Soccer	11	22.9	1.37	0.5 - 2.2
Basketball	9	21.9	1.35	0.5 - 2.3
Handball	9	16.9	1.14	0.4 - 1.9
Softball	1	9.0	0.26	-0.3 - 0.8
Athletics	1	5.0	0.16	-0.2 - 0.5
Total ankle ligament injuries	59	18.2*	0.99*	0.99 - 1.0*

These data include all sports, not just sports that presented ankle ligament sprains.

**Table 3:** Frequency, distribution, and rates of anterior cruciate ligament injuries in games and practice combined in 2013.

Anterior cruciate ligament injuries	Frequency	Percentage of all injuries	Injury rate per 1000 athleteexposures	95% Confidence interval
Basketball	3	7.3	0.45	-0.007 - 0.97
Handball	3	5.6	0.38	-0.06 - 0.82
Indoor Soccer	2	4.1	0.24	-0.1 - 0.6
Soccer	1	2.4	0.21	- 0.21 - 0.64
Athletics	1	5.0	0.16	-0.17 - 0.51
Total anterior cruciate ligament injuries	10	3.1*	0.16*	0.16 - 0.17*

* These data include all sports, not just sports that presented anterior cruciate ligament injuries.

## DISCUSSION

Currently, university sports in Brazil are nonprofessional. In contrast to the United States of America collegiate model, few universities provide players with scholarships, and most students have never previously been engaged in any competitive sport. Despite the amateur nature of Brazilian university sports, the game injury rate (13.13 injuries per 1000 A-Es, 95% IC = 10.3 - 15 or one injury every four games for a team of 20 participants) and the practice injury rate (4.47 injuries per 1000 A-Es, 95% IC = 3.9 - 5.1 or one injury every 10 practices for a team of 20 participants) of this population were very similar to the NCAA´s game injury rate (13.8 injuries per 1000 A-Es, 95% CI = 13.7 - 13.9) and practice injury rate (4.0 injuries per 1000 A-Es, 95% CI = 3.9 - 4.0) (10), respectively. In addition, this study found the game injury rate to be three times higher than the practice injury rate. Again, this trend is very similar to the NCAA, which found 3.5 times more injuries in games than in practice^10^ ^)^ Given the physicality, especially in games, of some NCAA sports that do not exist in Brazil, such as football and hockey, and the intense level of competition throughout the NCAA, we excepted to see a lower game per practice injury rate in Brazilian than in NCAA collegiate athletics. This is what was observed when comparing National Basketball Association (NBA) players with others from the Spanish Professional Basketball League, a less competitive basketball league. Factors such as longer games, less time of ball possession, and the dominance of man to man marking may be responsible for game injury rates in the NBA being twice as high as practice injury rates, compared to the Spanish Professional Basketball League in which the game injury rate is about one third of the practice injury rate.^11^

Data from the NCAA^10^ and the present study also support higher game injury rates compared to practice injury rates when examining just the sports played in both the USA and Brazil, such as soccer, volleyball, basketball, baseball and softball.

Although overall game, practice, and game per practice injury rates in this study were similar to those of the NCAA, the injury mechanism distribution was different between populations. While player contact is the major mechanism involved in game injuries in both this study (53%) and the NCAA (58%),^10^ most practice injuries in this study were non-contact (54%). This is in contrast to NCAA, where player contact is also the major mechanism of practice injuries (42%). In addition, non-contact injuries account for almost double the number of game injuries in this study (33%) compared to the NCAA (17%).^10^

One reason for the difference in injury mechanism distribution between this study and NCAA is the higher intensity and physicality of NCAA sports, games and practices. Furthermore, this study included individual sports such as swimming, athletics, tennis and table tennis, where an injury caused by player contact is rare. However, other aspects must be involved in the injury mechanism difference because many athletes in this study (83%) played contact sports, such as rugby, soccer and basketball. (Table 1)

Another important aspect behind the observed injury mechanism differences may be that student athletes from Brazil´s universities are generally much less physically trained than NCAA athletes. This may make them more prone to non-contact injuries, similar to the non-contact anterior cruciate ligament sprain predisposition of athletes who have worse neuromuscular control.^12^

Given most injuries among NCAA athletes occur from player contact, preventative measures from ISS have largely focused on rules and policies that promote more secure contact between players, such as the no spearing and no clipping rules instituted in football.^10^ In order for effective injury prevention strategies to be implemented at Brazil´s universities, one must consider that our needs are different from the NCAA, as advocated by van Mechelen et al.^7^

In this study, almost all sports had higher rates of injury in games than in practice. Rugby, the sport with the most contact between players, had the greatest difference: 6.72 times more injury in games (42.42 injuries per 1000 A-Es) than in practice (6.31 injuries per 1000 A-Es). Water polo had the lower difference: 1.05 times more injury in games (5.05 injuries per 1000 A-Es) than in practice (4.84 injuries per 1000 A-Es), followed by athletics, which had 1.19 times more injury in games (3.97 injuries per 1000 A-Es) than in practice (3.31 injuries per 1000 A-Es).

Judo was the only sport in which the opposite trend was observed: 1.46 times more injury in practice (13.47 injuries per 1000 A-Es) than in games (9.17 injuries per 1000 A-Es). Although higher game per practice injury rates in water polo and judo may be expected due to the large amounts of player contact, this was not observed. In water polo, the intense contact between players happens inside the water, which may lessen the injury risk. In judo, the higher incidence of injury in practice compared to games may be due to the time duration of each event. One practice session, which typically lasts around two hours, and one fight in a competition, which typically lasts around seven minutes, were both considered 1 A-E. 

Similar to the NCAA, our study found the lower limbs to be the most prevalent location for injury, accounting for 68% of injuries in games and 58.4% of injuries in practice (versus 54% in both practice and games in the NCAA).^10^ The lower limbs have also been found to be the most affected body part in various sports, such as rugby,^13^ ^,^ ^14^ football,^15^ soccer,^16^ ^,^ ^17^ basketball^11^ and volleyball,^18^ as well as in overuse injuries.^19^ This study found ankle ligament sprains and knee injuries to be most prominent, accounting for 18% and 11% of injuries, respectively. In the NCAA, ankle sprain is also the most common injury (15% of all injuries).^10^ In addition, other studies have found ankle sprain to be the most common injury in volleyball^20^ and basketball.^11^

A much smaller percentage of head and neck injuries were found in this study compared with the NCAA. These injuries account for 12.8% of game injuries and 9.8% of practice injuries in the NCAA^10^ but only accounted for 2.6% and 4.6% of injuries in this study, respectively. This is most likely due the NCAA's inclusion of football and hockey athletes, which have relatively high concussion rates. These sports were not played among our study population, and are not common in Brazil.

In this study, the ACL injury rate (0.16 injuries per 1000 A-Es, 95% CI = 0.16 - 0.17) and the ankle ligament sprain rate (0.99 injuries per 1000 A-Es, 95% CI = 0.99 - 1.0) were both statistically higher than in the NCAA (ACL injury rate of 0.15 injuries per 1000 A-Es, 95% CI = 0.14 - 0.15 and ankle ligament sprains rate of 0.83 per 1000 A-Es, 95% CI = 0.82 - 0.84).^10^ As non-contact injuries were most common in this study, and given the high incidence of non-contact ACL^21^ and ankle sprain^18^ injuries, it is easy to understand their increased occurrence. The School of Medicine and the School of Physical Education and Sports in this study also had a higher ACL injury rate (2.5% per person per year) than amateur athletes (0.03 - 1.62% per person per year) and a similar rate to professional athletes (0.15% - 3.67% per person per year),^22^ reinforcing a recent retrospective study that indicated ACL injury as one of the most common injuries in the same School of Medicine evaluated by the present study in the last 20 years.^23^

There are several examples of successful lower limbs injury prevention programs. A prospective controlled trial with more than 1100 women volleyball athletes showed a lower incidence of ankle sprain injuries in the intervention group, who performed proprioceptive exercises, compared with the control group (risk difference of 0.4 / playing 1000 hours, 95% CI = 0.1 - 0.7).^20^ In Santa Monica, California, more than 2900 female soccer players between the ages of 14 and 18 years substituted proprioceptive and neuromuscular exercises, focused on correct jumping and landing technique, in the place of a traditional warm up. An 88% decreasing ACL injuries was observed.^24^ These findings motivated American Academy of Orthopaedic Surgeons (AAOS) and the American College of Sports Medicine (ACSM) to support neuromuscular training programs in female soccer players to help prevent ACL injuries.^8^ ^)^ Summarizing, Brazilian university athletes are at great risk of sustaining non-contact injuries, such as ankle sprain and ACL injuries. Future injury prevention programs should focus on these types of injuries in order to be effective.

### Study limitation

Considering that this is a retrospective study, it may be susceptible to memory bias, which means that a subject may have listed just the injuries that he was able to remember. In contrast to the NCAA, the questionnaire was answered by the participants on their own, not by a team certified athletic trainer or physician. Finally, although the time of an athlete-exposure across most of sports (mainly collective sports) was almost the same (around two hours), it was not uniform among all athletes, especially considering competitions in individual sports like swimming or athletics.

## CONCLUSION

Brazilian university athletes are at great risk of sustaining non-contact injuries, such as ankle sprain and ACL injuries. Future injury prevention programs should focus on these types of injuries in order to be effective.
